# The Landscape of Cigar Marketing in Print Magazines from 2018–2021: Content, Expenditures, Volume, Placement and Reach

**DOI:** 10.3390/ijerph192316172

**Published:** 2022-12-02

**Authors:** Ollie Ganz, Olivia A. Wackowski, Stefanie Gratale, Julia Chen-Sankey, Zeinab Safi, Cristine D. Delnevo

**Affiliations:** 1 Rutgers Center for Tobacco Studies, Rutgers Biomedical and Health Sciences, New Brunswick, NJ 08901, USA; 2 Department of Health Behavior, Society and Policy, Rutgers School of Public Health, Piscataway, NJ 08854, USA

**Keywords:** cigars, tobacco, tobacco industry advertising

## Abstract

Cigar consumption has increased in the U.S. over the past decade, yet gaps remain in research on cigar promotion in print magazines. This study examines ad placement, volume, ad characteristics, and associated expenditures for cigars from 2018–2021, and readership data for magazines containing cigar ads. We merged content analysis data with Kantar Media data on magazine placement and expenditures and used magazine readership data from MRI-Simmons. The only brand in print magazines was Black & Mild (B & M), a top cigar brand in the U.S. There were 30 unique B & M magazine ads and 284 occurrences (i.e., appearances in magazines), translating to $46,504,578 in expenditures. All ads featured the word “enjoy/enjoyment” and a warning label. Filtered cigars were the most featured cigar type (75%) and sweets was the most featured flavor (78%). Nearly half of the publications in which B & M were advertised in have substantial Black/African American readership and were featured in publications with disproportionate young adult and Hispanic/Latino readership. This study identified tactics used in print advertising for a top cigar brand. Future research should examine how these tactics impact consumer perceptions. Findings of cigar ads reaching vulnerable populations may inform the FDA’s efforts to reduce health disparities through regulations and public education.

## 1. Introduction

Like cigarette smoking, cigar smoking can lead to a range of life-threatening conditions, such as cancer [1]. The cigar market is diverse and includes higher-end cigars such as premium cigars, as well as mass-merchandise cigars (i.e., filtered cigars and cigarillos), which are typically sold in convenience stores and comprise the majority of the cigar market [2]. Cigarillos may come with plastic or wooden tips affixed to the end of the product to facilitate their smoking, while filtered cigars closely resemble cigarettes in design, with a spongy filter, and can be perceived and used as cigarette substitutes. While cigarette consumption has seen a large decline in recent decades, mass-merchandise cigar product sales increased from $2.47 billion in 2009 to $3.27 billion in 2020 [3].

The popularity of cigars among vulnerable populations in the U.S. is well-documented. Indeed, cigar use, especially use of cigarillos and filtered cigars, is disproportionately common among Black/African American individuals [4,5,6,7,8]. Recent national data show that past 30-day cigar use is highest for Black/African American middle and high school students (3.3%) compared with their white (1.8%), Hispanic (1.7%), and multiracial (2.2%) counterparts [8]. Among adults, 4.6% of Black/African Americans reported using cigars “every day” or “some days,” compared with other racial/ethnic groups (0.9%−3.8%) [7]. Cigars—cigarillos in particular—are also popular among young people [6,9], with Black/African American young adults reporting the highest prevalence of smoking cigar products across racial/ethnic and age groups [5]. Cigar use is also disproportionately common among sexual minorities (i.e., lesbian, gay, bisexual and transgender [LGBT] individuals) [6].

Cigar products are marketed through a variety of channels, including magazines, social media, point-of-sale, websites, and musical events, and often feature appealing flavors, human models, music imagery and language, price promotions, and descriptors that highlight certain natural themes and language [10,11,12,13,14,15,16,17], which may increase product interest [18]. Another characteristic of cigar advertising is warning labels, which are important for communicating the risks of cigars to consumers [19]. In 2000, the seven largest cigar companies in the U.S. entered into an agreement with the Federal Trade Commission conceding to display one warning from a set of approved warning statements on cigar packaging and advertising (e.g., “SURGEON GENERAL WARNING: Cigar smoking can cause lung cancer and heart disease”) [20]. Numerous studies have examined the presence of warning labels on cigar packaging [21] and advertising across various channels, including social media [15,17,22] and the point-of-sale [23]. For example, an analysis of cigar brand Swisher Sweet’s Instagram posts found that about 50% of posts displayed a warning label [17]. When FDA extended its regulatory authority to include cigars with the 2016 Deeming Rule, they added additional requirements to cigar warning labels. While the FDA’s new requirements for cigar warnings on ads (increasing their size and prominence at the top of ads) were intended to take effect by August 2018, they were not enforced by FDA because of a successful legal challenge by cigar industry representatives, but, as recommended by the FDA, could be adopted voluntarily [24]. One study of littered cigar packs in Oakland, CA in 2019 found that 67% displayed warning labels that were compliant with the Deeming Rule’s voluntary guidance [25]. Little is known about the presence of warning labels (generally and voluntarily compliant ones) on cigar print advertising, which has important implications for communicating the health risks of cigars to consumers.

Cigar marketing is a contributing factor in the persistent disparities observed in cigar smoking prevalence, as previous research has shown that cigar marketing often targets Black/African American communities [26,27]. For example, numerous studies have found that cigar advertisements are disproportionately prevalent in stores in Black/African American neighborhoods [26]. There is also evidence of cigar marketing targeting young adults, Hispanic individuals, and sexual minorities [26,27,28,29]. The preponderance of research on cigar marketing is focused on the retail environment and online, including social media [26]. We are only aware of two studies in the past decade that examined cigar advertising in print magazines [10,30]. These studies are limited in that they did not capture ads for cigarillos or filtered cigars [30], which comprise almost the entirety of the cigar market [2], or they did not distinguish between ads across different channels (e.g., print magazine vs. online ad) [10].

Monitoring cigar marketing in print magazines is important for several reasons. First, print magazines have become increasingly popular. Between 2012 and 2020, the number of magazine readers who were 18 years and older steadily increased from 211 million to 229 million, with print magazines being the preferred magazine format despite a growing interest in online versions [31]. Furthermore, magazine advertising allows for tobacco companies to cast a wide net. Unlike direct-to-consumer marketing and brand-owned social media accounts that specifically target current tobacco users, magazines can reach a wide audience, including susceptible non-users [32]. On the flip side, print magazine ads can also be used to reach vulnerable populations. One study found that 36% of young adults (ages 18–24) in the U.S. read a print magazine in just a single week in 2018 [33]. Additionally, national data indicate that Black/African American youth and adults are more likely to read magazines than their white counterparts [34,35,36]. Moreover, a longitudinal study found that tobacco advertising in magazines contributes to the continuation of single-product and poly-product tobacco use among young adults [37]. Lastly, magazines are one of the few channels with trackable expenditures for cigar products and consumer consumption is more readily trackable for magazines through readership data, thus yielding a better understanding of reach.

This study aims to provide a comprehensive examination of print magazine ad placement, volume, characteristics, and associated expenditures for cigars over a four-year period (2018–2021). This study also examines print magazine readership data for publications that contain ads for cigars to understand if cigar companies are reaching vulnerable populations with their print magazine ads, like they have with other channels [26].

## 2. Materials and Methods

### 2.1. Data Sources

#### 2.1.1. Kantar

Print magazine ad data were obtained from Kantar Media for January 2018 to December 2021. Kantar monitors 209 print magazines and tracks ad placement and expenditures for ads in monitored publications. Tracked information includes the magazine in which the ad was found, date of printing, and estimated expenditures of running the ad. Each unique ad can have multiple observations, as it can appear in different magazines and/or different issues of the same magazine. During the study period, Black & Mild was the only cigar brand advertised in print magazines, with a total of 30 unique ads and 284 observations of these ads (52 in 2018, 50 in 2019, 82 in 2020 and 100 in 2021).

#### 2.1.2. MRI Simmons

Print magazine readership data came from the 2021 MRI-Simmons Spring Doublebase USA Survey, a national area, probability-based sample of US adults (*n* = 76,831), which is designed to assess media exposure/use and oversamples for young adults and Black/African American and Hispanic adults. Data were collected from March 2019 to May 2021.

### 2.2. Ad Content Analysis

#### Coding Procedures

We developed a coding guide based on an iterative review of the ads and a review of literature on cigar marketing, with input from all study authors. After the initial draft of the coding guide was developed, two staff each coded a small sample of ads. The coders and project lead (OG) then discussed all discrepancies until 100% agreement was reached, and the coding guide was revised accordingly. The full sample of advertisements was coded by two coders, and discrepancies were discussed by the coders and project lead. Agreement across codes was high (84–100%). The five categories of codes were: (1) product characteristics, (2) ad imagery and language, (3) descriptors, (4) warning label characteristics, and (5) miscellaneous.

Product characteristics. We coded all ads for the presence of any cigar pack, individual cigar(s), lit cigar(s), lighters, matches or ashtrays. We also coded for whether a “limited-edition” product was featured, a flavor was featured, what flavor(s) and the type of cigar (i.e., filtered, plastic tip, wood tip). Flavors and cigar types could be shown either on the pack/cigar or mentioned in the ad text.

Ad imagery and language. We coded for urban imagery (e.g., city views), alcohol-related imagery (e.g., glass of wine) and music-related imagery and text (the latter did not include Black & Mild flavors Jazz and Blues). We also coded for whether people were featured in the ad, their perceived sex and race, and whether the person was shown holding, smoking, or lighting a cigar.

Descriptors. We coded for the use of the following descriptors on the cigar packs or in the ad text: enjoy/enjoyment, taste, aroma, natural, smooth, and short.

Warning label characteristics. We also coded for whether a warning label was present on the ad (other than on a cigar pack pictured), warning label background color (i.e., white or black), whether the warning label was compliant with FDA’s voluntary guidance on label placement and size for cigar ads (i.e., top of ad, at least 20% of the ad) [24], and warning label contrast (i.e., low, medium, or high). Contrast was defined as how the background color of the label contrasted with the background color of the ad (e.g., a black-background label on an ad with a dark background was considered low contrast).

Miscellaneous. Lastly, we coded for the language of the ad (English or Spanish) and whether the ad featured a link or QR code to the brand website.

### 2.3. Analysis

The content analysis data and occurrence/expenditure data from Kantar were merged in Stata/MP 17.1 [38] for analysis. First, we examined overall occurrences and expenditures over time. We also examined the percentage of ad occurrences over time that featured a) warning labels that are compliant with FDA’s voluntary guidance and b) each cigar type. Next, we estimated the prevalence of each code among the unique ads (*n* = 30) and all the observations (*n* = 284), and we assessed the expenditures associated with each code and the proportion of total expenditures for each code. Lastly, we examined occurrences for, and expenditures associated with ads in each publication, as well as readership data for each publication. This included total number of readers, total expenditures, percent of readership and total number of readers who are young adults (ages 18–24), Black/African American, Hispanic, and LGBT. We compared readership data with population-level prevalence estimates of each group to identify whether readership across groups for each publication was disproportionately high. If the proportion of readership was greater than the proportion of a population for a given group, we considered readership to be disproportionately high among that group. These groups were selected due to research showing that they have historically been the targets of cigar marketing [26,27,29]. Readership data were weighted to be nationally representative.

## 3. Results

As noted above, all observed cigar ads were for Black & Mild. Black & Mild spent $46,504,578 on print magazine ads from 2018 through 2021; they spent the most money in 2018 ($16,046,406), followed by 2019 ($12,088,283). Expenditures for 2020 ($9,109,276) and 2021 ($9,260,613) were similar.

### 3.1. Ad Occurrences and Expenditures over Time

**Figure 1:**
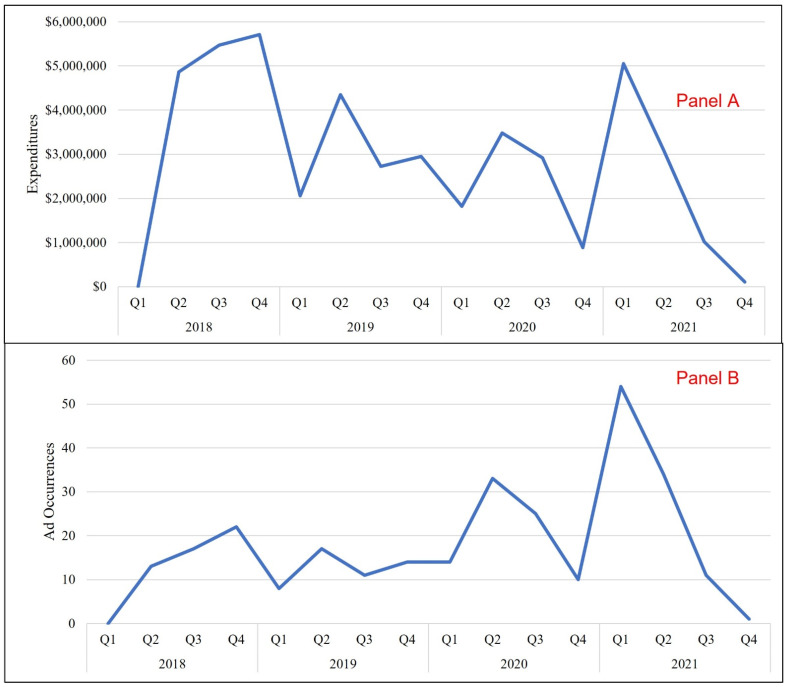
Print Magazine Advertising Expenditures (Panel A) and Occurrences (Panel B) for Black & Mild Cigars, 2018–2021.

Ad expenditures and occurrences by quarter can be found in Figure 1. Briefly, there were no expenditures or occurrences in Quarter (Q) 1 of 2018. Expenditures then increased, peaking in Q4 of 2018 and declining through Q1 of 2019. Expenditures then fluctuated for the remainder of the study period, declining to a low of $104,000 at the end of 2021. Ad occurrences also fluctuated during the study period but peaked in Q1 of 2021, followed by a rapid decline over the rest of the year.

### 3.2. Analysis of Cigar Type over Time

**Figure 2:**
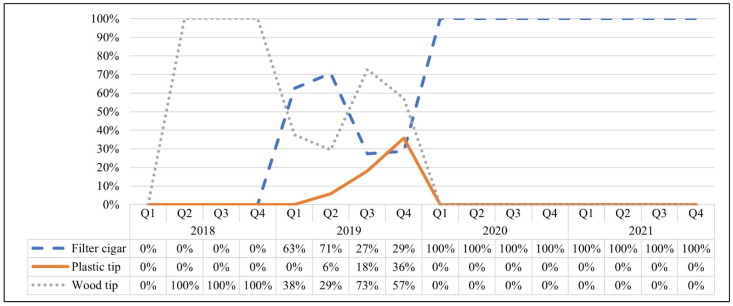
Percentage of Print Magazine Ad Occurrences for Black & Mild Featuring Each Cigar Type.

From Q2 to Q4 of 2018, all ad occurrences promoted wood tip cigars (Figure 2). Filtered cigars were not promoted until Q1 of 2019 and fluctuated through the remainder of the year. Beginning in Q1 of 2020, filtered cigars were featured in all ad occurrences and were the only cigar type featured. Plastic tip cigars were promoted briefly from Q2 of 2019 through Q4 of 2019 in a small percentage of ad occurrences.

### 3.3. Analysis of Voluntary Compliance with FDA Warning Labels over Time

**Figure 3:**
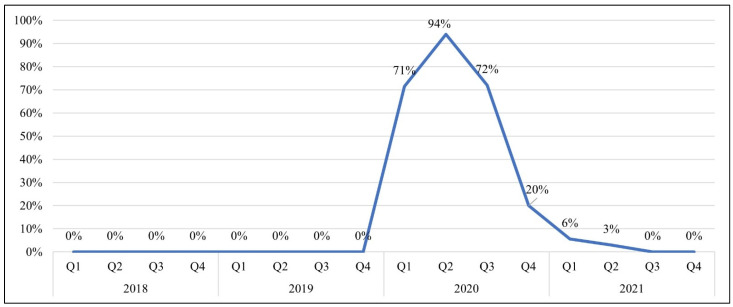
Percentage of Print Magazine Ad Occurrences for Black & Mild Featuring Voluntary Warning Labels.

No ad occurrences prior to 2020 featured a warning label that was voluntarily compliant with FDA’s size/placement guidelines (Figure 3). Compliance increased rapidly in Q1 of 2020 and peaked in Q2 of 2020 (94%), then declined quickly from 72% in Q3 of 2020 to 3% in Q2 of 2021. No ads featured a voluntarily compliant warning label in Q3 or Q4 of 2021.

### 3.4. Ad Occurrences and Expenditures, by Code

Table 1 presents findings from the analysis of ad content, occurrences, and expenditures. Below are key findings. All ad occurrences featured an individual cigar and most occurrences (91.5%) showed a cigar pack. Most ad occurrences (91.2%) featured a flavored cigar (totaling $40,894,292 in expenditures), with Sweets (77.8%) and Jazz (14.8%) being the most prominent flavors. Most occurrences promoted filtered cigars (75.5%), about one-quarter featured wood tip cigars (26.8%) and 2.8% featured plastic tip cigars. Likewise, expenditures were highest for ads featuring filtered ($25,016,753) and wood tip cigars ($21,211,145).

About one-third of the ad occurrences featured music-related imagery or language, totaling $18,814,669 over the study period. Urban imagery (21.5%; $6,961,541) and alcohol imagery (2.1%; $1,887,607) were less frequent. About one-third of occurrences featured a person (37.3%; $12,704,481) and 36.6% featured a person either holding, smoking, or lighting a cigar ($12,427,801). The presence of female (20.4%; $7,021,471) versus male (19.0%; $7,570,617) individuals was about the same. However, 36.6% of ad occurrences featured a Black/African American person ($12,427,801), while only 3.2% of occurrences featured a white person ($2,595,590). All ad occurrences featured the words “enjoy” or “enjoyment.” Less than 10% of occurrences featured the remaining descriptors examined (e.g., “aroma.”).

All occurrences featured a warning label, but only 22.9% of occurrences featured a warning label that was compliant with FDA’s voluntary guidance for warning size and placement ($7,061,055). Most occurrences contained warning labels with a white background (77.1%; $39,443,523) and 57% were coded as having high contrast with the ad ($30,612,114). Almost all ad occurrences were in English (98.9%; $46,004,278) and a total of 5.6% of ad occurrences featured a link or QR code to the brand’s website ($5,111,484).

### 3.5. Readership Data for Publications Featuring Cigar Ads

Black & Mild ads were identified in 26 publications, with readership figures ranging from approximately 2 to 11.9 million (Table 2). Almost all publications have disproportionate Black/African American readership, who comprise 13.4% of the US population [39], including 12 magazines that have Black/African American readerships of at least 20%—notably about 34% for two titles. Four magazines had young adult (ages 18–24) readerships of at least 15% (young adults comprise 12% of the US population) [40], including Rolling Stone, which alone included over 20 ad occurrences over the study period and is comprised of 20% young adult readership. Hispanic/Latino individuals comprise 18.5% of the US population [39] and several publications contained disproportionately high Hispanic/Latino readership, including People in Español (73.5%), OK Weekly (27.1%) and Elle (27.1%). With LGBT individuals comprising 7.1% of the US population [41], only one publication contained notably high LGBT readership—GQ (24.0%). However, we were unable to obtain readership data for Out, which is an LGBT publication.

## 4. Discussion

Our study is the first to report on the content, placement, and expenditures for cigar advertising in print magazines. Over a four-year period, we found that only one cigar brand advertised in consumer print magazines—Black & Mild. Black & Mild is the top-selling cigar brand in the U.S. and increased in popularity from 2009 to 2020 [3]. Its advertising dominance in this channel may be indicative of its strong advertising efforts in general and could related to the brand’s performance. Many of our findings align with previous research, including studies showing that cigar ads feature flavored cigars [16] and music imagery and language [10].

Interestingly, expenditures and occurrences indicating prominent codes in the ads did not always align. For example, 75.5% of occurrences promoted filtered cigars, yet when looking at expenditures, only 53.8% of expenditures were associated with ads promoting filtered cigars. This likely reflects the varying costs of advertising in different publications, as magazines with greater readership are likely more expensive to advertise in. This finding highlights the importance of examining both ad occurrences and expenditures, as one cannot assume that they tell the same story about a brand’s advertising strategy.

Our finding that most ad occurrences featured the brand’s cigar pack is noteworthy given the importance of brand recognition for purchasing at the point-of-sale, and the emerging literature on the impact of cigar packaging on consumer perceptions and intentions [42,43,44,45,46]. For example, one experimental study of cigarillo smokers found that elements of Black & Mild packaging, including the logo and brand name, presence of a price promotion, and pack color, influenced positive perceptions of the brand, such as smelling nice, containing high quality tobacco, reduced harshness, as well as intentions to purchase the product [42]. Therefore, the nearly ubiquitous presence of Black & Mild packaging in their magazine ads may reinforce and augment positive associations with the brand, above and beyond the content and language in the ad itself.

Our study found that the phrases “enjoy” or “enjoyment,” which have been used frequently in premium cigar advertising [47], were featured in all ads. These phrases may be used to remind smokers of the pleasure and satisfaction they feel from smoking, as well as elicit positive beliefs about the brand and enhance perceived value of the product [48]. Indeed, satisfying psychological and emotional needs has been a common tactic used by cigarette companies in the past to target vulnerable populations [49]. While not common, other descriptors were featured in the ads, including natural and smooth, which comprised about 10% and 9% of ad expenditures, respectively. Use of these terms may imply reduced harm and chemical exposure from using the products [16] and may contribute to product appeal among younger populations [50].

Most ad occurrences (75.5%) and about half of all ad expenditures were devoted to filtered cigars and from 2020–2021, filtered cigars were the only cigar type featured in Black & Mild ads. Filtered cigars look nearly identical to cigarettes and are typically sold in larger pack sizes (e.g., packs of 5 or 20), whereas other types of cigars, like cigarillos, are usually sold in packs of five or less [3]. Historically, filtered cigars (also known as little cigars) have been promoted by tobacco companies in response to increasingly stringent policies and high taxes on cigarettes, as well as growing concern from the public regarding the negative health effects of cigarette smoking [51]. A review of previously secret tobacco industry documents found that filtered cigars were in fact designed for and marketed toward cigarette smokers [51], and a recent analysis of marketing for Cheyenne, a popular filtered cigar brand, found that the product was promoted as being similar to cigarettes [22]. While we cannot say for certain why Black & Mild’s magazine ads placed a strong emphasis on promoting their filtered cigars, it could be an attempt to lure cigarette smokers who may be looking for a cheaper alternative to cigarettes. Many ads for filtered cigars featured the phrase “shift your enjoyment,” which could be a subtle way of suggesting that consumers shift their enjoyment from cigarettes to filtered cigars. Another potential reason for the emphasis on filtered cigars in recent Black & Mild magazine ads is the forthcoming ban on characterizing flavors in cigars, which John Middleton Co. (owner of Black & Mild), likely anticipated before the announcement of the ban in April 2021 [52]. The cigarillo market is heavily flavored [3], including Black & Mild’s current lineup of wood and plastic tip cigarillos, which are available in six different flavors. This contrasts with the brand’s filtered cigar, which is only available in one flavor, according to the brand’s website (www.blackandmild.com, accessed on 15 June 2022). As such, Black & Mild may be pushing their largely unflavored, filtered cigar in anticipation of the ban on characterizing flavors in cigars.

In terms of warning labels, we did not observe any adoption of the new voluntary labeling guidelines until the second quarter of 2020. Furthermore, after the key lawsuit decision in July 2020 ruled in favor of the industry and the warning requirements were not upheld, Black & Mild’s voluntary compliance with the warning provisions dropped and then ceased. These findings suggest that the company was aware of the new FDA warning guidelines and capable of implementing them, but was not interested in doing so if not required. This underscores the need for cigar-specific research on warning label effectiveness to support relevant future policy and implementation efforts [53,54,55,56].

Taken together, findings on readership data as well as ad content illustrate a concerning pattern. Specifically, nearly half of the publications in which Black & Mild advertised have substantial Black/African American readership. Correspondingly, over 10 times as many Black & Mild ad occurrences in the study period featured Black/African American people than white people. Considering the disproportionate prevalence and burden of cigar smoking on Black/African American individuals [4,5,6], the brand’s selection of ad imagery as well as specific publications speak to a concerning and potentially intentional targeting of minority populations. Readership data also show that Black & Mild ads were featured in publications with disproportionate young adult and Hispanic/Latino readership, suggesting that targeted marketing of cigarettes toward these groups, which is documented in the literature [27], may also extend to cigar products.

Our study has limitations. First, although print magazines are still the preferred format among most readers, our study did not include ads or readership data for digital versions of the publications, and therefore we may not have captured additional exposure to cigar ads via digital issues. Those who prefer print magazines over digital tend to be older and of higher socioeconomic status (SES) [31], therefore our findings from MRI-Simmons may skew older and higher SES compared with data on readership of digital magazines. Additionally, Kantar does not monitor all print magazines, or specialty cigar publications (e.g., Cigar Aficionado), and therefore the number of occurrences and expenditures are likely an underestimate. Furthermore, data from MRI-Simmons rely on self-report and therefore may be subject to recall bias [57]. Data from MRI-Simmons is also restricted to adults, so we are unable to capture readership among youth. Lastly, our analysis is focused on advertising in the U.S. and is not necessarily generalizable to cigar advertising in other countries.

## 5. Conclusions

The influence of advertising on tobacco use can be exacerbated by communication inequalities, which are differences in the ability to access, understand, and act upon health information [58]. Findings from our study demonstrate that vulnerable populations, Black/African American adults in particular, are exposed to excess risk-promoting messages for cigar products in print magazines. Advertisers traditionally use a “media mix” approach to marketing where they integrate messaging across numerous platforms [58]. While this study only focuses on one of these platforms—print media—other research demonstrates that Black/African Americans are exposed to disproportionately high levels of cigar marketing via other channels as well, including the point-of sale [26]. These inequalities across the media mix likely contribute to elevated cigar smoking among this population [7,8].

Overall, this study fills an important gap in the literature by helping to demystify a piece of the cigar marketing mix, by providing analysis of the content, expenditures, and reach of cigar marketing via print magazines in the U.S. Our study also exposes future research needs related to cigar marketing. Indeed, research is needed to understand how the strategies identified in our study influence consumer perceptions and intentions among both users and non-users, and among different population groups (e.g., Black/African Americans, young adults). Furthermore, as the tobacco marketplace continues to change and as cigar companies adjust their marketing strategies in response to the forthcoming ban on characterizing flavors, continued surveillance of cigar marketing in print magazines, as well as other channels, is warranted. Lastly, findings from our study may inform the FDA’s educational efforts related to communicating the risks of cigars to consumers.

## Figures and Tables

**Table 1:** Characteristics of Unique Ads, Ad Occurrences and Corresponding Expenditures for Black & Mild Print Magazine Ads, 2018–2021.

	Proportion of Unique Ads (*n* = 30)	Percentage of Total Occurrences (*n* = 284)	Expenditures	Percentage of Total Expenditures
**Product characteristics**	% (*n*)	% (*n*)	$	%
Pack shown	73.3 (22)	91.5 (260)	39,986,029	86.0
Individual cigar(s) shown	100 (30)	100 (284)	46,504,578	100
Lit cigar(s) shown	30.0 (9)	40.1 (114)	15,729,703	33.8
Lighter, matches or ashtray shown	13.3 (4)	17.6 (50)	11,557,387	24.8
Limited edition product featured	20.0 (6)	7.4 (21)	6,199,479	13.3
Flavored cigar shown ^µ^	96.7 (29)	91.2 (259)	40,894,292	87.9
Flavors featured				
Sweets	66.7 (20)	77.8 (221)	28,732,037	61.8
Wine	13.3 (4)	9.9 (28)	8,507,698	18.3
Jazz	16.7 (5)	14.8 (42)	14,151,404	30.4
Casino	6.7 (2)	5.3 (15)	3,715,284	8.0
Deluxe	6.7 (2)	1.1 (3)	511,146	1.1
Blues	13.3 (4)	2.8 (8)	1,214,989	2.6
Cigar type featured ^µ^				
Filtered	60.0 (18)	75.5 (206)	25,016,753	53.8
Plastic tip	16.7 (5)	2.8 (8)	1,106,896	2.4
Wood tip	36.7 (11)	26.8 (76)	21,211,145	45.6
**Ad imagery and language**				
Urban imagery	10.0 (3)	21.5 (61)	6,961,541	15.0
Music-related imagery/language	33.3 (10)	35.2 (100)	18,814,669	40.5
Alcohol imagery	3.3 (1)	2.1 (6)	1,887,607	4.1
People shown in ad	26.7 (8)	37.3 (106)	12,704,481	27.3
Person shown holding/smoking/lighting cigar	26.7 (8)	36.6 (104)	12,427,801	26.7
Perceived sex of people in ad				
Male	23.3 (7)	19.0 (54)	7,570,617	16.3
Female	10.0 (3)	20.4 (58)	7,021,471	15.1
Can’t tell	0 (0)	0 (0)	0	0
Perceived race of people in ad				
White	10.0 (3)	3.2 (9)	2,595,590	5.6
Black	26.7 (8)	36.6 (104)	12,427,801	26.7
Can’t tell	0 (0)	0 (0)	0	0
**Descriptors**				
Enjoy/enjoyment	100 (30)	100 (284)	46,504,578	100
Taste	13.3 (4)	8.4 (24)	4,920,595	10.6
Aroma	13.3 (4)	7.0 (20)	4,611,203	9.9
Natural	6.7 (2)	4.6 (13)	4,792,414	10.3
Smooth	16.7 (5)	7.0 (20)	3,937,063	8.5
Short	6.7 (2)	2.8 (8)	2,670,912	5.7
**Warning labels**				
Warning label present	100 (30)	100 (284)	46,504,578	100
Warning label background color				
White label with black text	76.7 (23)	77.1 (219)	39,443,523	84.8
Black label with white text	23.3 (7)	22.9 (65)	7,061,055	15.2
Warning label contrast w/ad background				
Low	23.3 (7)	20.4 (58)	6,724,789	14.5
Medium	26.7 (8)	22.5 (64)	9,167,675	19.7
High	50.0 (15)	57.0 (162)	30,612,114	65.8
Voluntary compliance with FDA’s guidance for warning size/location ^¶^	23.3 (7)	22.9 (65)	7,061,055	15.2
**Miscellaneous**				
Language of ad				
English	93.3 (28)	98.9 (281)	46,004,278	98.9
Spanish	6.7 (2)	1.1 (3)	500,300	1.1
Link or QR code to brand website featured	13.3 (4)	5.6 (16)	5,111,484	11.0

Note: Expenditures over the study period (2018–2021) totaled $46,504,578. ^µ^ Could be shown on the pack/cigar or in ad text. ^¶^ Placement of warning label on top of ad, at least 20% of the ad.

**Table 2:** Total Occurrences, Expenditures and Magazine Readership for Black & Mild Print Magazine Ads, 2018–2021.

Magazine	Number of Occurrences	Total Expenditures ($)	Total Readership	Young Adults (18–24)		Black/African American		Hispanic/Latino		Lesbian, Gay, Bisexual or Transgender	
				%	Population Total	%	Population Total	%	Population Total	%	Population Total
In Touch Weekly	43	2,395,917	2,975,000	10.9%	324,000	21.8%	649,000	19.4%	576,000	4.3%	127,000
Life & Style Weekly	41	936,567	µ	µ	µ	µ	µ	µ	µ	µ	µ
Star	23	3,768,680	3,307,000	11.1%	366,000	25.0%	827,000	18.0%	595,000	5.4%	180,000
Rolling Stone	21	4,332,825	7,226,000	20.0%	1,442,000	16.7%	1,205,000	25.0%	1,803,000	6.7%	487,000
OK Weekly	21	1,982,840	2,267,000	11.4%	259,000	22.1%	501,000	27.1%	614,000	5.2%	119,000
TV Guide	18	2,730,100	7,010,000	9.0%	629,000	23.6%	1,657,000	16.5%	1,156,000	4.4%	307,000
Entertainment Weekly	14	3,344,627	8,574,000	10.0%	859,000	21.7%	1,858,000	18.3%	1,571,000	6.7%	578,000
Sports Illustrated	12	5,201,200	11,930,000	13.0%	1,554,000	20.5%	2,440,000	15.2%	1,817,000	2.8%	335,000
US Weekly	10	3,032,850	7,362,000	13.6%	1,000,000	18.6%	1,371,000	18.5%	1,359,000	6.6%	487,000
GQ	10	2,481,231	3,952,000	15.3%	604,000	34.1%	1,349,000	21.9%	867,000	10.4%	410,000
People in Español	9	1,117,000	5,755,000	10.3%	594,000	11.0%	631,000	73.5%	4,228,000	3.0%	173,000
Men’s Journal	8	2,275,770	2,469,000	10.2%	251,000	23.3%	575,000	17.0%	419,000	6.6%	162,000
Time	7	2,072,182	11,507,000	11.7%	1,342,000	16.5%	1,902,000	17.7%	2,041,000	5.8%	670,000
Popular Mechanics	7	1,809,050	4,620,000	5.5%	256,000	9.8%	452,000	12.5%	576,000	3.5%	164,000
Out	7	415,343	µ	µ	µ	µ	µ	µ	µ	µ	µ
Esquire	6	1,600,398	2,061,000	9.3%	192,000	33.9%	698,000	20.6%	424,000	7.0%	144,000
Golf Magazine	5	1,595,580	3,905,000	6.2%	242,000	9.6%	376,000	8.8%	343,000	¶	¶
Popular Science	4	556,880	µ	µ	µ	µ	µ	µ	µ	µ	µ
In Style	3	1,076,500	5,189,000	8.9%	461,000	22.2%	1,154,000	19.7%	1,021,000	4.8%	247,000
Elle	3	742,960	3,922,000	15.0%	587,000	26.2%	1,027,000	27.1%	1,062,000	6.2%	244,000
Car and Driver	2	763,965	5,424,000	6.5%	352,000	11.6%	629,000	16.2%	879,000	3.1%	166,000
Athlon Sports & Life	2	598,800	µ	µ	µ	µ	µ	µ	µ	µ	µ
Travel + Leisure	2	531,600	5,071,000	4.6%	233,000	14.6%	741,000	13.4%	680,000	3.8%	195,000
Vogue	2	424,292	8,121,000	18.7%	1,521,000	22.7%	1,845,000	26.8%	2,174,000	8.5%	687,000
Golf Digest	2	406,277	3,529,000	4.6%	162,000	8.0%	284,000	5.8%	203,000	1.5%	52,000
Wired	2	311,144	2,537,000	12.7%	322,000	11.0%	280,000	17.4%	442,000	6.9%	174,000

µ—not measured in database. ¶—cell size too small to report. Data source: 2021 MRI-Simmons Spring Doublebase USA Survey (*n* = 76,831).

## Data Availability

Not applicable.
